# Cortical Stimulation Induces Excitatory Postsynaptic Potentials of Inferior Colliculus Neurons in a Frequency-Specific Manner

**DOI:** 10.3389/fncir.2020.591986

**Published:** 2020-10-26

**Authors:** Jiyao Qi, Zizhen Zhang, Na He, Xiuping Liu, Caseng Zhang, Jun Yan

**Affiliations:** Department of Physiology and Pharmacology, Hotchkiss Brain Institute, Cumming School of Medicine, University of Calgary, Calgary, AB, Canada

**Keywords:** auditory cortex, corticofugal system, postsynaptic potentials, inferior colliculus, mouse

## Abstract

Corticofugal modulation of auditory responses in subcortical nuclei has been extensively studied whereas corticofugal synaptic transmission must still be characterized. This study examined postsynaptic potentials of the corticocollicular system, i.e., the projections from the primary auditory cortex (AI) to the central nucleus of the inferior colliculus (ICc) of the midbrain, in anesthetized C57 mice. We used focal electrical stimulation at the microampere level to activate the AI (ES_AI_) and *in vivo* whole-cell current-clamp to record the membrane potentials of ICc neurons. Following the whole-cell patch-clamp recording of 88 ICc neurons, 42 ICc neurons showed ES_AI_-evoked changes in the membrane potentials. We found that the ES_AI_ induced inhibitory postsynaptic potentials in 6 out of 42 ICc neurons but only when the stimulus current was 96 μA or higher. In the remaining 36 ICc neurons, excitatory postsynaptic potentials (EPSPs) were induced at a much lower stimulus current. The 36 ICc neurons exhibiting EPSPs were categorized into physiologically matched neurons (*n* = 12) when the characteristic frequencies of the stimulated AI and recorded ICc neurons were similar (≤1 kHz) and unmatched neurons (*n* = 24) when they were different (>1 kHz). Compared to unmatched neurons, matched neurons exhibited a significantly lower threshold of evoking noticeable EPSP, greater EPSP amplitude, and shorter EPSP latency. Our data allow us to propose that corticocollicular synaptic transmission is primarily excitatory and that synaptic efficacy is dependent on the relationship of the frequency tunings between AI and ICc neurons.

## Introduction

The auditory cortex sends large numbers of descending projections to most auditory nuclei in the thalamus, midbrain, and low brainstem (Weedman and Ryugo, 1996; Druga et al., 1997; Winer et al., 1998, 2001; Rouiller and Welker, 2000; Schofield and Coomes, 2005). These corticofugal projections comprise a feedback system that enables cortex-oriented modulation or control of the neural processing of incoming sound information (Syka and Popelár, 1984; Suga et al., 2000; Jen et al., 2002; Xiong et al., 2009; Bajo et al., 2010; Bajo and King, 2013; Terreros and Delano, 2015; Suga, 2020).

Following the pioneering work by Suga and his colleagues (Yan and Suga, 1996; Suga et al., 1997; Zhang et al., 1997), a surge of studies over the last quarter-century has established a highly specific corticofugal function. Specifically, cortical neurons implement differential modulation of the auditory responses of subcortical neurons depending on the functional relationship of cortical and subcortical neurons, facilitation when cortical and subcortical neurons have similar tunings, and suppression when they have different ones (Suga, 2020). This cortex-oriented modulation is seen across various domains i.e., frequency, amplitude, and time (Yan and Suga, 1996; Ma and Suga, 2001; Yan and Ehret, 2002; Zhou and Jen, 2007), various processing centers i.e., thalamus, midbrain, and cochlear nucleus (Zhang and Suga, 2000; Zhou and Jen, 2000; Luo et al., 2008; Liu et al., 2010) and various species i.e., bats, gerbils and mice (Zhang et al., 1997; Zhou and Jen, 2000; Sakai and Suga, 2002; Yan and Ehret, 2002). To date, little is known about the synaptic mechanism underlying the corticofugal system and its highly specific modulation.

Recognized as a convergence and/or integration center, the inferior colliculus (IC) of the midbrain is often chosen as the target for corticofugal studies (Druga et al., 1997; Zhang et al., 1997; Gao and Suga, 1998; Zhou and Jen, 2000, 2007; Yan and Ehret, 2002; Bajo and King, 2013). The direct projections from the primary auditory cortex (AI) to the central nucleus of the inferior colliculus (ICc) are tonotopically organized (Feliciano and Potashner, 1995; Saldaña et al., 1996; Bajo and Moore, 2005; Lim and Anderson, 2007; Markovitz et al., 2013), providing an anatomical basis of highly specific corticocollicular modulation, at least in the frequency domain. Physiological studies show that focal electrical stimulation of the AI (ES_AI_) facilitates the responses of ICc neurons to the frequency that is tuned by the stimulated AI neurons, whereas it suppresses responses to the frequencies that are not tuned by the stimulated AI neurons (Zhang and Suga, 2000; Zhou and Jen, 2000; Yan and Ehret, 2002). Yet another consideration, inactivation of the entire auditory cortex with muscimol (GABA_A_R agonist) reduces the responses of ICc neurons to all frequencies in a non-specific manner (Zhang and Suga, 1997; Yan and Suga, 1999). This finding suggests that direct AI-to-ICc projections are likely excitatory in general, which allows tonic support of auditory responses in ICc neurons. A question raised here is which postsynaptic potential (PSP) can be induced by ES_AI_: excitatory PSP (EPSP), inhibitory PSP (IPSP), or both. Another important issue is the possibility that ES_AI_-evoked PSPs exhibit frequency specificity.

This study focusses on AI-to-ICc PSPs and examines the ES_AI_-evoked changes in the membrane potentials of ICc neurons in anesthetized C57 mice. The membrane potentials of ICc neurons were recorded by whole-cell current-clamp. We found that the majority of ICc neurons exhibited EPSPs after ES_AI_. ES_AI_ also induced IPSPs in a few ICc neurons, but only with the use of strong stimulus current. ES_AI_-evoked EPSPs exhibited a lower threshold, shorter latency, and greater amplitude when the stimulated AI neurons and recorded ICc neurons had similar frequency tunings i.e., characteristic frequencies (CFs).

## Materials and Methods

**Figure 1:**
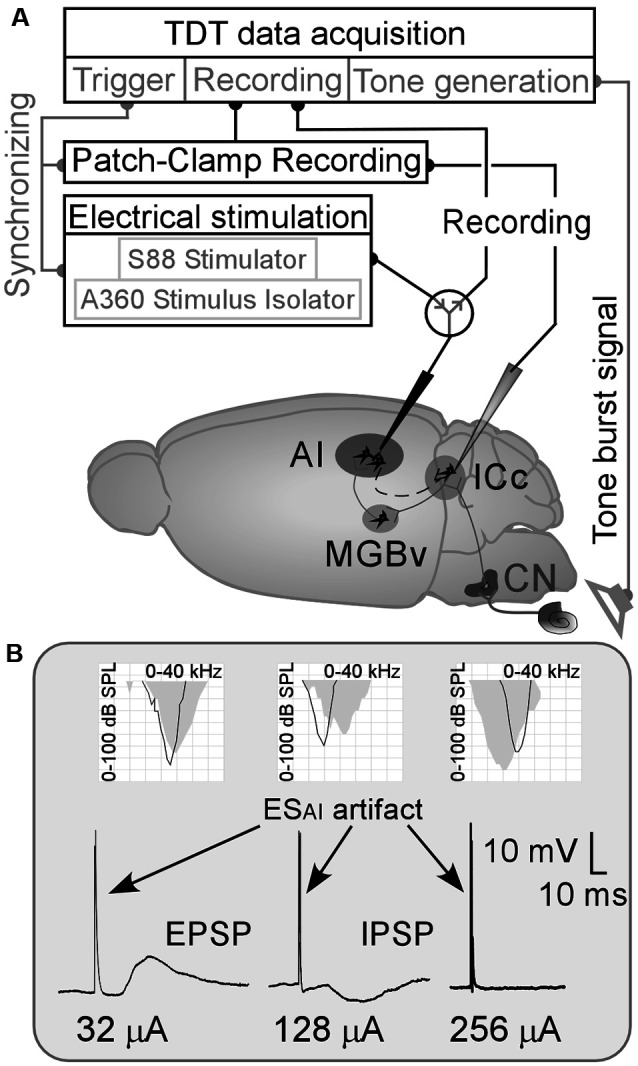
**(A)** Schematic drawing of the experimental approach/mouse brain. AI, primary auditory cortex; CN, cochlear nucleus; ES_AI_, focal electrical stimulation of the AI; ICc, central nucleus of the inferior colliculus; MGBv, ventral division of the medial geniculate body; 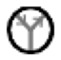: a switch between recording system and stimulation system. **(B)** Examples of ES_AI_-evoked changes in membrane potentials of ICc neurons and their frequency tunings (AI—gray areas and the ICc—black curves in the grid fields). EPSP and IPSP, excitatory and inhibitory postsynaptic potentials (PSPs).

Our study examined 46 female C57 mice aged 4–7 weeks and weighing 15–25 g. Animal use was following the Canadian Council on Animal Care, and our protocol (AC14–0215) was approved by the Animal Care Committee at the University of Calgary. A schematic diagram of our experimental approach is shown in Figure 1A.

### Animal Preparations

Mice were anesthetized throughout the surgery and physiological experiments by intraperitoneal injection. We used a mixture of ketamine (85 mg/kg, Bimeda-MTC Animal Health Inc., Canada) and xylazine (15 mg/kg, Bimeda-MTC Animal Health Inc., Canada). Additional doses of ketamine and xylazine (17 and 3 mg/kg, respectively) were given to maintain anesthesia if the animals showed any response to tail pinching. A custom-made head holder was used to fix the mouse’s head by clamping between the palate and nasal bones. The Bregma and lambda of the skull were aligned in the horizontal plane. The scalp, subcutaneous tissue, and muscle were then removed to expose the skull. Two holes measuring 2 mm in diameter were made with a dental drill to expose the left primary auditory cortex (AI, 2.2–3.6 mm posterior to the Bregma, 4.0–4.5 mm lateral to the midline) and the left central nucleus of the inferior colliculus (ICc, 0.5–2.0 mm posterior to the lambda, 0.5–2.0 mm left to the midline). The exposed dura was gently removed. A feedback-controlled heating pad was used to maintain the body temperature of the mouse at ~37°C during surgery and all experiments. The electrophysiological studies were conducted in an echo-attenuated chamber with electromagnetic shielding and soundproofing.

### Acoustic Stimulation

A 20 ms-long pure tone burst (5 ms for both rise- and fall-times) was used for acoustic stimulation. Tone bursts were digitally generated and converted to analog signals by an RZ6 MULTI I/O processor (Tucker-Davis Technologies, Inc., Gainesville, FL, USA). The analog signals were sent to a digital attenuator and then to a loudspeaker (MF1, Tucker-Davis Technologies., Gainesville, FL, USA) positioned at 45° and 15 cm away from the right ear of the mouse. The speaker output (tone amplitude) was calibrated at the same position using a condenser microphone (Model 2520, Larson-Davis Laboratories, USA) and a microphone preamplifier (Model 2200C, Larson-Davis Laboratories, USA). The tone amplitude was expressed as dB SPL (re. 20 μPa). Frequencies and amplitudes of tone bursts were changed manually or digitally *via* BrainWare data acquisition software (Tucker-Davis Technologies, Inc., Gainesville, FL, USA). A frequency-amplitude scan (FA-scan) was used to sample the receptive field (frequency tuning curve) of a recorded neuron. The frequency varied from 3 to 40 kHz with 1 kHz increments and the amplitude from 5 to 85 dB SPL with 5 dB increments. To sample a reliable frequency tuning curve of a single ICc neuron, the FA-scan was repeated three times and the frequency/amplitude of tone for each FA-scan was randomly altered using BrainWare software.

### Recording and Focal Electrical Stimulation of the AI

The responses of AI neurons were recorded using a tungsten electrode (~2 MΩ impedance), which was placed perpendicularly to the surface of the left auditory cortex and connected to a recording system *via* a headstage (Tucker-Davis Technologies, Inc., Gainesville, FL, USA). During the electrode penetration, tone-evoked action potentials were commonly identified in layers III/IV of the cortex (approximately 300–600 μm below the brain surface). After 5–8 penetrations, the location of AI was identified according to the tone-evoked response properties. The frequency tuning curves of AI neurons were first sampled by using an FA-scan and stored using BrainWare software. The same electrode was then advanced to a depth of about 700–800 μm below the brain surface to layer V and its connection was switched from a recording system to a stimulating system. Since the AI is organized in columns, the CFs of the AI in layer V and layers III/IV are identical (Abeles and Goldstein, 1970; Shen et al., 1999; Moerel et al., 2018). An indifferent electrode was placed on the brain surface next to the stimulating electrode. Electrical pulses (0.2 ms long, negative, monophasic square wave), generated by a Grass S88 stimulator (Astro-Medical, Inc., West Warwick, RI, USA) and an A360 constant current isolator (WPI, Inc., Sarasota, FL, USA), were delivered to deep layers of the AI through the tungsten electrode (i.e., ES_AI_).

### Whole-Cell Patch-Clamp Recording in the ICc

Glass pipettes (Sutter Instrument, Novato, CA, USA) were pulled to construct a glass electrode with a tip diameter of ~1 μm (7–12 MΩ in impedance) for patch-clamp recording. The electrodes were filled with an intracellular solution of 125 mM K-gluconate, 20 mM KCl, 10 mM Na_2_ phosphocreatine, 4 mM MgATP, 0.3 mM Na_2_GTP, 0.5 mM EGTA, and 10 mM HEPES (7.25 pH and 290 mOsm). A silver wire inserted into the electrode was connected to the MultiClamp 700B amplifier (Molecular Device, Sunnyvale, CA, USA) through a headstage. The bioelectrical signals from the electrode were filtered by a 4 kHz low-pass filter using a MultiClamp 700B amplifier and digitized using the DigiData1550 (Molecular Device, Sunnyvale, CA, USA) at a sampling rate of 10 kHz. They were recorded and stored using Clampex 10.4 data acquisition software (Molecular Device, Sunnyvale, CA, USA). BrainWare data acquisition software (Tucker-Davis Tech., Inc., Alachua, FL, USA) was also used to record these signals to tag the parameters of acoustic stimulation on to tone-evoked events.

For whole-cell patch-clamp recording, the interelectrode pressure of the glass pipette electrode was set at 200–300 mbar and the MultiClamp 700B was set to voltage-clamp mode. The electrode was first positioned perpendicularly in the left ICc at about 400 μm from the brain surface and then advanced 1 μm per step using a digital manipulator. During the stepped penetration, a positive square voltage pulse (amplitude of 10 mV and a duration of 10 ms) was continuously delivered to monitor the electrode tip impedance using the Clampex data acquisition software. Confirmation of the electrode tip contacting the membrane of a neuron was typically indicated by a sharp increase (~20%) in tip impedance. Once contact was established, the interelectrode pressure was released. A successful seal of the electrode tip on the neuronal membrane as indicated by a giga-ohm tip impedance. A negative pressure (20–30 mbar) was then applied to break the cell membrane. When attaining whole-cell patch configuration, the whole-cell capacitance was compensated completely, and the series resistance (20–60 MΩ) was compensated by 50–80%. The MultiClamp 700B amplifier was then switched to the whole-cell current-clamp mode in which the electrode capacitance was neutralized, and the current holding was set to 0 pA mode (He et al., 2017).

### Experimental Protocol and Data Acquisition

Once the tungsten electrode was positioned in the AI, the following procedures were performed. First, the responses of AI neurons to tones with various frequencies and amplitudes were recorded (FA-scan). This established the CF of AI neurons. Second, ICc neurons were patched. Third, resting membrane potentials of given ICc neurons were recorded. Fourth, changes in membrane potentials of given ICc neurons were recorded in response to the FA-scan and a repetitive tone at the CF (20 dB above the MT) and 50 ms intervals. The recording was allowed to continue when a neuron exhibited sharp tuning and no adaptation. Last, the membrane potentials of ICc neurons were recorded before and after the ES_AI_. The stimulus current was set to 2^x^ μA. The value of x ranged from 1 to 10.

### Data Processing and Statistics

The data acquired were processed and analyzed using a custom-made SoundCode program and a Clampfit 10.4 program (Molecular Device, Sunnyvale, FL, USA). The frequency tunings of AI and ICc neurons were measured using SoundCode software and the changes in membrane potential of ICc neurons in response to tone and ES_AI_ were measured using Clampfit software.

Based on the frequency tuning curves, the minimum threshold (MT) was defined as the lowest dB SPL that was able to induce noticeable responses to tone across various frequencies. The CF was the frequency at the MT. Based on the relationship between the CFs of the recorded ICc neurons and the stimulated AI neurons, the ICc neurons were sorted into two groups: physiologically matched and unmatched neurons. If the CFs of AI and ICc neurons were similar (≤1 kHz), the neurons were labeled matched neurons; if the CFs were >1 kHz, they were labeled unmatched neurons.

Stimulus-evoked events were determined by the change in the membrane potential that was 20% larger than the averaged fluctuation of the baseline. The EPSP was a positive-going wave and the IPSP was a negative-going wave. The EPSP waveforms of ICc neurons were characterized using amplitude, latency, 50% duration, and a rising slope. The amplitude of an EPSP waveform was determined by the range between the baseline and the peak of the waveform. The latency was measured as the time from stimulus onset to the EPSP onset (the crossing point of the baseline to the upward slope line of the waveform). The 50% duration was the time when the membrane potential exceeded the 50% mark of the EPSP amplitude. The rising slope was defined as the EPSP amplitude divided by the time from the onset to the peak of a given EPSP waveform.

Data were expressed as means ± SD. The ANOVA test was used to compare the differences between groups of data, and a *p*-value of less than 0.05 was considered statistically significant.

## Results

Eighty-eight ICc neurons were successfully patched in 46 mice. The resting membrane potentials (RMPs) of these ICc neurons are shown in Supplementary Table 1 and the CFs and MTs of sampled ICc neurons and corresponding AI neurons are shown in Supplementary Table 2. The CFs and MTs of these AI and ICc neurons fell within the central hearing range of C57 mice (Zhang et al., 2005; Heffner and Heffner, 2007; Luo et al., 2009). The ES_AI_ induced noticeable changes in membrane potential in 42 neurons as shown in Figure 1B (left and middle). The 40 neurons that had exhibited no membrane potential changes following the ES_AI_ up to 256 μA (Figure 1B, right). The RMPs of ICc neurons and frequency tunings (CFs and MTs) of both AI and ICc neurons were not significantly different between the “response” and “no response” groups (Supplementary Tables 1, 2). Two neurons experienced a loss of signal resulting in an interrupted recording. The data from the “no response” group as well as from the neurons with interrupted recordings were then excluded.

Out of 42 neurons showing changes in membrane potential, 36 ICc neurons exhibited depolarization (excitatory PSPs—EPSPs, Figure 1B, left), and six neurons exhibited hyperpolarization (inhibitory PSPs—IPSPs, Figure 1B, middle) following ES_AI_. The frequency tunings between AI and ICc neurons were different based on these two samples. These samples (Figure 1B, left and middle) also show an important feature of EPSP/IPSP induction; the current for EPSP is far lower than that for IPSP. On average, the threshold current of the ES_AI_ for EPSP induction ranged from 6 to 64 μA (38.17 ± 17.90 μA, *n* = 36) while that for an IPSP ranged from 96 to 128 μA (106.67 ± 15.08 μA, *n* = 6). ES_AI_-evoked IPSPs were not specific to the tuning relationship of AI and ICc neurons. These findings were different from the ES_AI_-evoked EPSPs as presented below. Considering the small sample size of IPSP data, the discussion focuses mostly on ICc neurons exhibiting ES_AI_-evoked EPSPs.

### Dynamic Range of ES_AI_-Evoked EPSPs

**Figure 2:**
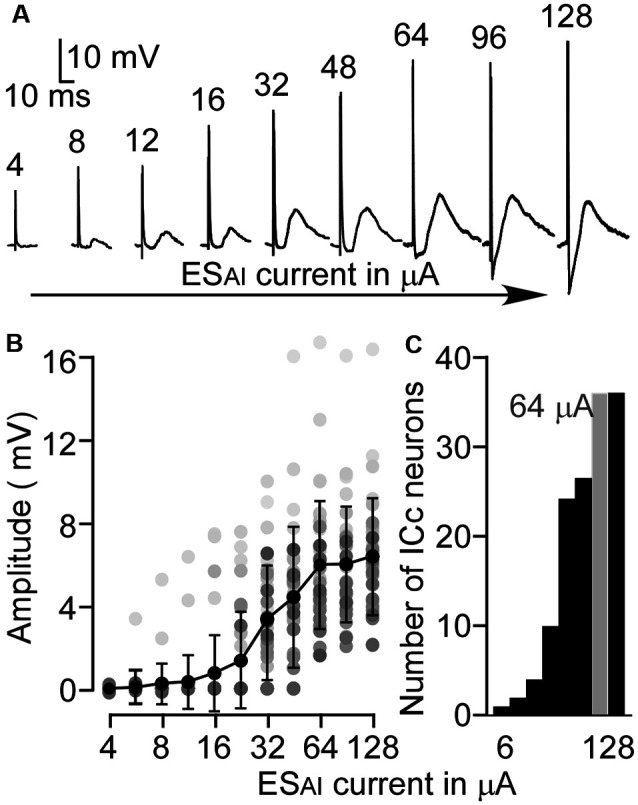
The amplitude-to-intensity function of ES_AI_-evoked EPSPs. **(A)** An example of the ES_AI_-evoked EPSPs of an ICc neuron following ES_AI_ at a series of current levels. **(B)** Summarized amplitude-to-intensity function of ES_AI_-evoked EPSPs (*n* = 36). Gray dots represent the sample distributions at different stimulus levels. **(C)** Histogram of the number of neurons showing EPSP in response to ES_AI_. In this study, 64 μA (gray) was the lowest, optimal level because it enabled noticeable EPSP in all 36 ICc neurons.

The EPSP amplitudes of ICc neurons evoked by ES_AI_ were tested by a series of currents. As shown in Figure 2A, the ES_AI_ induced noticeable EPSPs in a neuron when the stimulus current was 8 μA or higher. EPSP amplitude gradually increased in response to increases in current. Figure 2B shows the EPSP amplitudes of ICc neurons as the function of ES_AI_ currents in a range from 4 to 128 μA. On average, the EPSP amplitude exhibited a sharper increase when the current of ES_AI_ ranged from 24 to 64 μA and rarely increased from 64 to 128 μA. Figure 2C shows the number of ICc neurons exhibiting EPSPs in responses to stimulus currents. Since the level of 64 μA evoked reliable EPSPs in all 36 ICc neurons, we used this data to characterize the ES_AI_-evoked EPSPs.

### Characterization of ES_AI_-Evoked EPSPs of ICc Neurons

**Figure 3:**
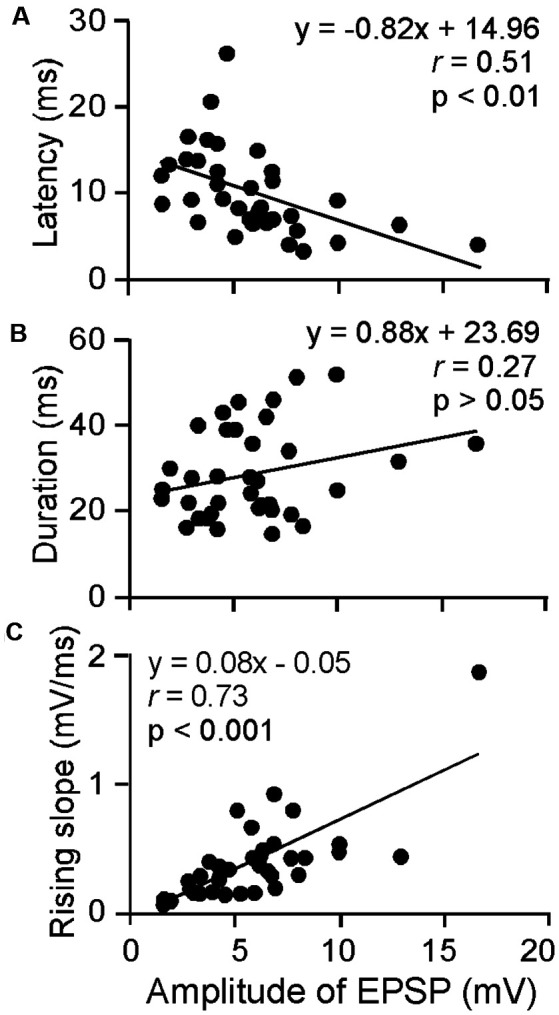
Scatter plotting of latency **(A)**, 50% duration **(B)** and rising slope **(C)** as the function amplitude of ES_AI_-evoked EPSPs of ICc neurons. At the 64 μA stimulation level, the latency and rising slope were significantly correlated to amplitude whereas duration was insignificant. The solid lines represent the regression.

The amplitude, latency, rising slope, and 50% duration were measured for the ES_AI_-evoked EPSPs of ICc neurons; the amplitude appeared to correlate with the latency, rising slope, and 50% duration. For example, a larger amplitude was associated with shorter latency, longer duration, and a larger rising slope. As shown in Figure 3, the latency and rising slope were significantly correlated to the amplitude (*r* = −0.51, *p* < 0.01, Figure 3A and *r* = 0.73, *p* < 0.001, Figure 3C). However, the 50% duration was poorly correlated to the amplitude (*r* = 0.27, *p* > 0.05, Figure 3B). At the level of 64 μA, the EPSP amplitude ranged from 1.59 to 16.66 mV (5.91 ± 3.05 mV, *n* = 36). The latency ranged from 3.30 to 26.30 ms (10.14 ± 4.92 ms, *n* = 36). The 50% duration ranged from 14.80 to 51.80 ms (28.92 ± 10.44 ms, *n* = 36). The rising slope ranged from 0.07 to 1.87 mV/ms (0.40 ± 0.32 mV/ms, *n* = 36).

### ES_AI_-Evoked EPSP vs. Frequency Tunings

As described above, ICc EPSPs evoked by ES_AI_ at the 64 μA level showed obvious variation from neuron to neuron. Previous studies using extracellular recording consistently demonstrate frequency-specific corticofugal modulation of the auditory responses of ICc neurons (Yan and Suga, 1998; Zhang and Suga, 2000; Jen et al., 2002; Yan and Ehret, 2002). ES_AI_ induces the facilitation of tone-evoked auditory responses when the difference in frequency tunings (CFs) between stimulated AI neurons and recorded ICc neurons is equal to or smaller than 1 kHz (physiologically matched neuron). Suppression is induced when the CF difference of stimulated AI neurons and recorded ICc neurons is larger than 1 kHz (physiologically unmatched neuron). We next analyzed if and how ES_AI_-evoked EPSPs were associated with the frequency tunings (CFs) of AI and ICc neurons.

**Figure 4:**
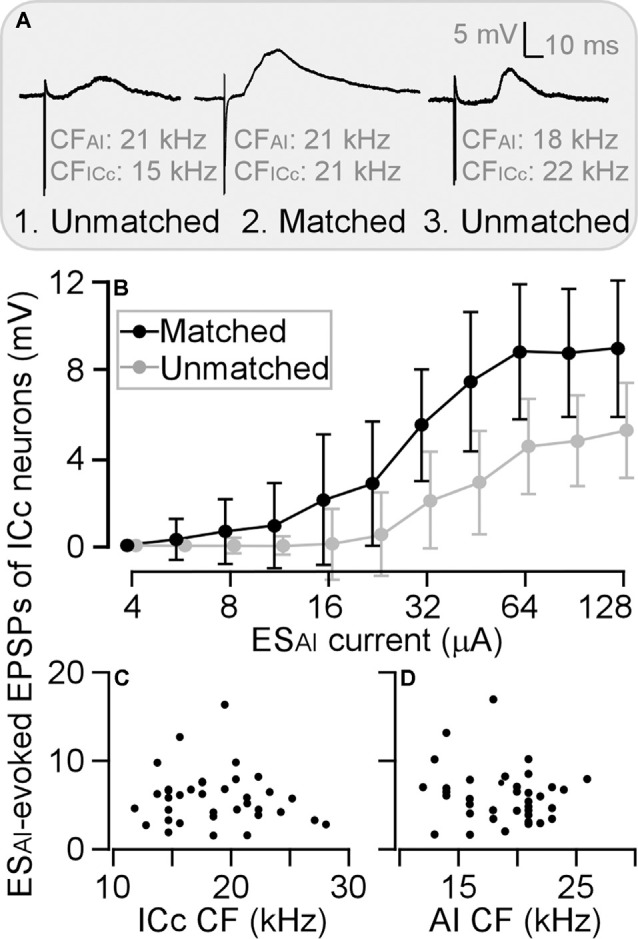
Differences in the amplitude of ES_AI_-evoked EPSPs between matched and unmatched ICc neurons. Three examples show greater EPSP of matched neurons compared with those of unmatched neurons at the stimulation level of 64 μA **(A)**. This difference is also shown by amplitude-to-intensity function **(B)**. However, the ES_AI_-evoked EPSPs exhibit no relation to CFs of ICc neurons **(C)** or AI neurons **(D)**. AI, primary auditory cortex; CF, characteristic frequency; EPSP, excitatory postsynaptic potential; ES_AI_, focal electrical stimulation of the AI; ICc, central nucleus of the inferior colliculus.

To be consistent with previous studies (Yan and Ehret, 2002; Wu and Yan, 2007; Luo et al., 2008; Liu et al., 2015), we sorted ICc neurons into two groups: a matched group when the CF difference between the recorded ICc neurons and stimulated AI neurons was 1 kHz or less (Figure 4A) and an unmatched group when the CF difference was larger than 1 kHz. Three examples are shown in Figure 4A. The ICc neuron in Figure 4A was tuned to 15 kHz and its corresponding AI neuron tuned to 21 kHz; the ICc CF was 6 kHz lower than AI CF. The ICc neuron in Figure 4A tuned to 22 kHz and its corresponding AI neuron tuned to 18 kHz; the ICc CF was 4 kHz higher than AI CF. These two neurons were sorted as physiologically unmatched neurons. In contrast, the CF of a neuron in Figure 4A was 21 kHz, identical to that of the corresponding AI neuron. This neuron was therefore sorted as a physiologically matched neuron. The EPSP amplitude of these ICc neurons exemplifies the efficacy of ES_AI_. The ES_AI_-evoked EPSP was greater in matched ICc neurons than in unmatched neurons. Examining the EPSP amplitude as the function of ES_AI_ current demonstrated that the matched neurons (*n* = 12) had a steeper slope than unmatched neurons (*n* = 24); ES_AI_ evoked larger EPSPs of ICc neurons at all current levels (Figure 4B). Similar to previous findings, ES_AI_-evoked EPSPs were only associated with the CF difference between the recorded ICc and stimulated AI neurons; no correlation was observed between the ES_AI_-evoked EPSPs and the CFs of either the recorded ICc (Figure 4C) or stimulated AI neurons (Figure 4D).

As illustrated in Figure 4B, ES_AI_-evoked EPSPs were larger in matched than unmatched ICc neurons at all tested stimulus intensities. We further compared the threshold currents of evoking EPSPs induction, 64-μA-level EPSP latency, and 64-μA-level EPSP amplitude between matched and unmatched ICc neurons.

**Figure 5:**
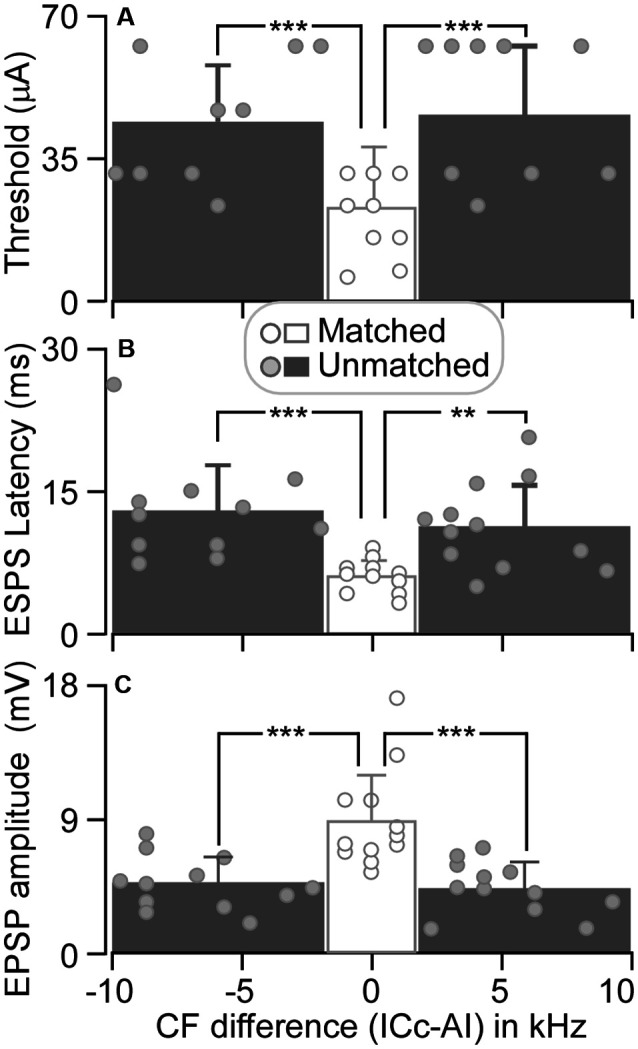
The threshold induced EPSP **(A)**, EPSP latency **(B)**, and amplitude **(C)** are plotted as the function of CF difference between ICc and AI neurons. Histograms show the averaged values of ICc neurons that had similar CFs to (middle), lower than (left), and higher than (right) those of AI neurons. **p < 0.005; ***p < 0.001.

The threshold of matched ICc neurons ranged from 6 to 32 μA, with an average of 23.17 ± 9.22 μA (*n* = 12). For unmatched ICc neurons, the threshold ranged from 24 to 64 μA (44.67 ± 15.13 μA, *n* = 12) when AI CFs were higher than ICc CFs and from 24 to 64 μA (46.67 ± 17.54 μA, *n* = 12) when AI CFs were lower than ICc CFs (Figure 5A). The ES_AI_ threshold current in matched neurons was significantly lower than the threshold current in unmatched neurons, i.e., ICc CF < AI CF (*p* < 0.001) and ICc CF > AI CF (*p* < 0.001).

At a stimulus level of 64 μA, the latency and amplitude of ES_AI_-evoked EPSPs of ICc matched neurons exhibited larger variation, whereas the latency and amplitude were different between matched and unmatched neurons (circles in Figures 5B,C). The EPSP latencies of matched neurons were shorter than those of unmatched neurons. On average, the latency of matched neurons was 6.03 ± 1.72 ms (*n* = 12). The latency of matched neurons was significantly shorter than 13.05 ± 4.83 ms (CF: ICc < AI, *n* = 12, *p* < 0.001) and 11.33 ± 4.41 ms (CF: ICc > AI, *n* = 12, *p* < 0.005) of unmatched neurons (columns in Figure 5B). Similarly, the EPSP amplitudes of matched neurons were different from those of unmatched neurons. The EPSP amplitude of matched neurons was 8.77 ± 3.12 mV (*n* = 12). This result was significantly greater than 4.62 ± 1.72 mV (CF: ICc < AI, *n* = 12, *p* < 0.001) and 4.33 ± 1.68 mV (CF: ICc > AI, *n* = 12, *p* < 0.001) of unmatched neurons (columns in Figure 5C).

## Discussion

The auditory cortex modulates the subcortical responses to sound stimulation in a frequency-specific manner (Zhang et al., 1997; Yan and Suga, 1998; Yan and Ehret, 2002; Zhou and Jen, 2007). As for the synaptic mechanism of such specific corticofugal modulation, this study substantiates three fundamental characteristics in the AI-to-ICc pathway, i.e., ES_AI_-evoked PSPs of ICc neurons. First, AI-to-ICc synaptic transmission is primarily excitatory since the majority of ICc neurons exhibited EPSPs following ES_AI_ (Figure 2). Second, the inhibitory synaptic transmission may be involved in corticofugal modulation although the ES_AI_-evoked IPSPs were limited to only a few ICc neurons and required a much larger stimulus intensity (high threshold, Figure 1B). Finally, corticofugal synaptic transmission appears to occur in a frequency-specific manner as the ES_AI_-evoked EPSPs were significantly different between matched and unmatched ICc neurons (Figures 4A,B, 5).

### Corticofugal Excitation

The primary EPSP of the AI-to-ICc pathway is consistent with previous findings in several lines of study. Biochemical and immunochemical studies demonstrate that the neurotransmitter of corticocollicular synapses is glutamate (Feliciano and Potashner, 1995; Ito and Oliver, 2010). The *N*-methyl-D-aspartate receptor (NMDAR) and the metabotropic glutamate receptor (mGluR) that mediate corticofugal excitatory transmission have been demonstrated in different sensory systems and different species including rats (Malmierca and Nuñez, 2004), guinea pigs (McCormick and von Krosigk, 1992), cats (Scharfman et al., 1990; Rivadulla et al., 2002), and monkeys (Montero and Wenthold, 1989). Physiological studies show that the inactivation of the entire auditory cortex reduces the auditory responses of ICc neurons, suggesting excitatory effects in general (Zhang and Suga, 1997; Yan and Suga, 1999).

Previous findings, together with our data, allow us to glean an understanding of direct corticofugal pathways. Cortical neurons send direct glutamatergic projections to subcortical (i.e., ICc) neurons. When cortical neurons are active, corticofugal terminals release glutamate that acts on the postsynaptic NMDAR and mGluR, leading to postsynaptic depolarization and modulating the excitability of postsynaptic neurons.

One phenomenon of long ES_AI_-evoked EPSP latency must be noted. The latency was related to stimulus intensity; the greater the stimulus intensity, the shorter the EPSP latency (Figures 2B, 3A). At the 64 μA level, the ES_AI_-evoked EPSP was about 6 ms in matched neurons, the latency reported here is far longer than previously reported (1–1.4 ms, Mitani et al., 1983). We assume that one explanation for this may be the difference in stimulus intensity although this information is not provided by the Mitani group. Yet another explanation might be the NMDAR that mediates the late EPSP component. Studies in different preparations show that the NMDAR-mediated latency can range from 3 to 6 ms (Shirokawa et al., 1989; Armstrong-James et al., 1993; Metherate and Ashe, 1994). Another consideration is multiple synaptic transmission; the AI-to-ICc pathway, even for matched neurons, may have multiple synaptic transmission as discussed below.

### Corticofugal Inhibition

The neurotransmitter of corticofugal projections is glutamate, which acts on NMDAR and mGluR of postsynaptic neurons. Since GABAergic terminals are not found in the projections from the auditory cortex to the ICc (Feliciano and Potashner, 1995), the ES_AI_-evoked ICc IPSP must have an indirect effect. In other words, AI neurons innervate local (collicular) GABAergic neurons that in turn innervate the neurons recorded in the ICc (Stebbings et al., 2014).

In the inferior colliculus (IC), GABAergic neurons are widely distributed; the percentage of GABAergic neurons in the ICc appears to be slightly larger than that in the external cortex of the IC (ICx), a non-lemniscal subdivision (Oliver et al., 1994; Merchán et al., 2005). Up to 25% of ICc neurons are GABAergic neurons that are large in soma size and evenly distributed across the tonotopic organization (Merchán et al., 2005; Wong and Borst, 2019), implicating that no less than 25% of ICc neurons recorded in this study could be GABAergic. These histological features support our findings that the ES_AI_ was also able to evoke the IPSP of ICc neurons. When compared to the ES_AI_-evoked EPSP, two notable differences emerge. That is, IPSP was observed in fewer ICc neurons, and additionally, the threshold for inducing IPSP was much higher. Although a detailed analysis was not performed in this study due to limited sample size, ES_AI_-evoked IPSPs favor the previous findings that ES_AI_ inhibits the tone-evoked responses of physiologically unmatched subcortical neurons (Yan and Ehret, 2002; Luo et al., 2008).

### Frequency-Dependence of ES_AI_-Evoked ICc EPSPs

Previous studies demonstrated that ES_AI_ induces highly frequency-specific modulation of the auditory responses of ICc neurons in the same species (Yan and Ehret, 2002; Yan et al., 2005) and in other species such as mustached bats, big brown bats, and gerbils (Gao and Suga, 1998; Yan and Suga, 1998; Sakai and Suga, 2002; Zhou and Jen, 2007; Bajo and King, 2013). The frequency-specificity appears to suggest a universal law of corticofugal modulation. In this study, an important finding is that the ES_AI_-evoked ICc EPSPs also obey this law; EPSP induction was related to the CF difference between AI and ICc neurons (Figure 5). Our findings provide an initial understanding of a synaptic basis for the interpretation of frequency-specific corticofugal modulation of tone-evoked responses of ICc neurons.

Two themes derived from our findings of the ES_AI_-evoked EPSPs are worthy of our attention. One is how the ES_AI_-evoked EPSP is dependent on the difference in frequency tunings (i.e., CFs) between AI and ICc neurons. The other is how the frequency-specificity of ES_AI_-evoked EPSPs can be converted into tone-evoked firing behavior of ICc neurons as observed in previous studies.

Concerning the frequency-dependency of the ES_AI_-evoked EPSPs, three explanations are possible. The first relates to the damped propagation of electrical current within the brain. This means that AI neurons at a distance from the stimulus electrode (ref. to unmatched neurons) require a greater stimulus current to achieve a similar level of activation than those positioned near the electrode tip (ref. to matched neurons). Considering the tonotopic organization of the AI-to-ICc pathway (Huffman and Henson, 1990; Saldaña et al., 1996; Druga et al., 1997; Winer et al., 2001; Lim and Anderson, 2007; Bajo et al., 2010), the damped propagation of electrical current likely explains why the ES_AI_-evoked EPSPs had a lower threshold (Figure 5A) and higher amplitude (Figure 5C) in matched than in unmatched ICc neurons. However, this interpretation may be flawed as the ES_AI_-evoked EPSPs of matched neurons exhibited a shorter latency than those of unmatched neurons (Figure 5B). Also, we found that ES_AI_-evoked EPSPs were similar between ICc neurons with CFs lower and higher than AI CF (Figure 5). With damped propagation, these EPSP properties should be different because the tonotopic organization of the auditory system is based on a logarithmical scale. For example, the affecting distance of 64 μA is about 500 μm (Ranck, 1975). If a stimulus electrode were placed at the 17 kHz area of the AI, we would expect that our stimulus current would affect the range from the 11 kHz area (low-frequency end) to the 28 kHz area (high-frequency end) according to the tonotopic organization in the AI of C57 mice (Zhang et al., 2005). A second explanation for the frequency-dependency may be the neural “spread” of the ES_AI_ effect due to intracortical excitatory projections (Sutter et al., 1999; Metherate et al., 2005). In other words, the AI neurons distant from the electrode tip may be activated or modulated by the neural inputs from the AI neurons in the vicinity of the electrode tip. This interpretation is also supported by our recent finding that ES_AI_ induces frequency-specific changes in auditory responses of other AI neurons in a linear scale under thalamic inactivation (Kong et al., 2018). A third explanation may involve intra-collicular interactions, including the inhibitory projections from the ICx to ICc, as discussed above.

As for how ES_AI_-evoked EPSPs can be converted to the frequency-specific changes in tone-evoked firing behavior of ICc neurons, the significance of postsynaptic glutamate receptor NMDAR and mGluR must be considered. It is well established that glutamate binding to NMDAR depolarizes postsynaptic neurons through cation influx and facilitates the input-specific responses (synaptic plasticity) of postsynaptic neurons (Furukawa et al., 2005; Li and Tsien, 2009). mGluR is a metabotropic receptor; it’s binding with glutamate leads to changes in the excitability of postsynaptic neurons through the modulation of other ion channels (Chu and Hablitz, 2000; Gabriel et al., 2012). Our study suggests that corticofugal modulation of postsynaptic excitability through NMDAR/mGluR must have a significant impact on the responses of postsynaptic neurons to ascending inputs (i.e., tone-evoked inputs); greater corticofugal EPSP translates to a greater impact on the auditory responses of postsynaptic neurons (i.e., ICc neurons).

We propose that the ES_AI_-evoked EPSP, through NMDAR and mGluR, facilitate the tone-evoked EPSP of ICc neurons, particularly when descending and ascending inputs are temporally close to each other. Furthermore, the strength of the corticofugal modulation depends on the amplitude of the ES_AI_-evoked EPSP. Both proposals merit future investigation.

### Possible Pathways of AI-to-ICc Transmission

Based on the above discussions, the AI-to-ICc transmission must involve both mono- and multi-synaptic transmission, and the pathways for matched neurons and unmatched neurons must be different.

In theory, the AI-to-ICc pathway can be mono-synaptic for matched neurons. However, the pathway should involve many multi-synaptic transmissions because the ES_AI_ at 64 μA can stimulate a group of neurons in the vicinity of the stimulus electrode through intracortical projections. In this scenario, the recorded EPSP of ICc neurons may consist of multiple synaptic inputs from a group of AI-to-ICc projections. Consequently, the EPSP properties should be dependent on the strength and timing of these inputs. This likely explains why the correlation of EPSP amplitude and duration is relatively poor (Figure 3B).

The significantly longer EPSP latency of unmatched ICc neurons suggests indirect (multi-synaptic) AI-to-ICc pathways. A possible pathway is that the stimulated AI neurons, *via* intracortical connections, activate other AI neurons that in turn act on collicular neurons through corticofugal projections. A well-tested pathway proposed by Jen and group (Jen et al., 1998) is an AI-ICx-ICc pathway; AI neurons activate ICx GABAergic neurons that in turn inhibit the ICc neurons. This pathway is supported by several important findings. First, corticofugal neurons more extensively innervate the ICx (Huffman and Henson, 1990). Second, ICx neurons send GABAergic fibers to the ICc (Merchán et al., 2005). Third, the ES_AI_ with a larger current increases the tone-evoked responses of ICx neurons but decreases those of ICc neurons in a non-frequency-specific manner (Jen et al., 1998). Fourth, the electrical stimulation of the ICx inhibits the tone-evoked responses of ICc neurons (Jen et al., 1998). Fifth, the ICx-inhibition of tone-evoked ICc responses can be eliminated by local application of bicuculline (an antagonist for GABA_A_ receptor) to the ICc (Jen et al., 2001). Last, our recorded data of the ES_AI_-evoked IPSP was only observed with a strong current, i.e., 106.67 ± 15.08 μA.

## Conclusion

This study reveals for the first time that ES_AI_ primarily evoked EPSPs of ICc neurons i.e., AI-to-ICc excitatory synaptic transmission, in a frequency-specific manner. Such frequency-specific effects may rely on intracortical and/or intra-collicular circuits. Inhibitory circuits from ICx to ICc may also contribute to the frequency-specific variation of the ES_AI_-evoked EPSPs. Our findings provide an initial understanding of the synaptic basis for frequency-specific corticofugal modulation of subcortical auditory information processing.

## Data Availability Statement

All datasets presented in this study are included in the article/Supplementary Material.

## Ethics Statement

The animal study was reviewed and approved by Animal Care Committee at the University of Calgary.

## Author Contributions

JY supervised this study. JQ contributed to all experiments, data analysis, and manuscript writing. ZZ provided technical support. NH and XL contributed to the electrophysiological experiments. CZ contributed to data processing and manuscript editing. All authors contributed to the article and approved the submitted version.

## Conflict of Interest

The authors declare that the research was conducted in the absence of any commercial or financial relationships that could be construed as a potential conflict of interest.
